# Angiotensin II-Related Activation of Scleral Fibroblasts and Their Role on Retinal Ganglion Cell Death in Glaucoma

**DOI:** 10.3390/ph16040556

**Published:** 2023-04-06

**Authors:** Si-Eun Oh, Jie-Hyun Kim, Hee-Jong Shin, Seong-Ah Kim, Chan-Kee Park, Hae-Young Lopilly Park

**Affiliations:** Department of Ophthalmology, Seoul St. Mary’s Hospital, College of Medicine, The Catholic University of Korea, Seoul 06591, Republic of Korea

**Keywords:** angiotensin II, extracellular matrix, systemic hypotension, glaucoma

## Abstract

We identify the angiotensin II (AngII)-associated changes in the extracellular matrix (ECM) and the biomechanical properties of the sclera after systemic hypotension. Systemic hypotension was induced by administering oral hydrochlorothiazide. AngII receptor levels and ECM components in the sclera and biomechanical properties were evaluated based on the stress–strain relationship after systemic hypotension. The effect of inhibiting the AngII receptor with losartan was determined in the systemic hypotensive animal model and the cultured scleral fibroblasts from this model. The effect of losartan on retinal ganglion cell (RGC) death was evaluated in the retina. Both AngII receptor type I (AT-1R) and type II (AT-2R) increased in the sclera after systemic hypotension. Proteins related to the activation of fibroblasts (transforming growth factor [TGF]-β1 and TGF-β2) indicated that transformation to myofibroblasts (α smooth muscle actin [SMA]), and the major ECM protein (collagen type I) increased in the sclera after systemic hypotension. These changes were associated with stiffening of the sclera in the biomechanical analysis. Administering losartan in the sub-Tenon tissue significantly decreased the expression of AT-1R, αSMA, TGF-β, and collagen type I in the cultured scleral fibroblasts and the sclera of systemic hypotensive rats. The sclera became less stiff after the losartan treatment. A significant increase in the number of RGCs and decrease in glial cell activation was found in the retina after the losartan treatment. These findings suggest that AngII plays a role in scleral fibrosis after systemic hypotension and that inhibiting AngII could modulate the tissue properties of the sclera, resulting in the protection of RGCs.

## 1. Introduction

Glaucoma is characterized by progressive loss of the retinal ganglion cells (RGCs), and the main cause is elevated intraocular pressure (IOP) [1]. Various factors affect the glaucomatous process, and one of the most important is the biomechanical properties of the supporting tissue within the optic nerve head (ONH), including the lamina cribrosa and the sclera. The components of the extracellular matrix (ECM) and resulting biomechanical factors of the supporting tissues through which the RGC axons pass are thought to determine the degree of RGC damage in glaucoma. Therefore, investigating the cause and factors affecting the ECM changes and the biomechanical properties of the lamina cribrosa and sclera are important to understand the pathogenesis of glaucoma. We have previously detected a difference in the ECM components of the lamina cribrosa and sclera between Western and Asian eyes [2]. Epigenetic control of the ECM genes differs by race, and this seems to contribute to the racial difference in the type of glaucoma. In addition, systemic vascular dysregulation is an important feature of glaucoma other than elevated IOP [3,4,5]. Systemic arterial hypotension, orthostatic hypotension, unstable or fluctuating systemic blood pressure (BP), Raynaud’s phenomenon, and migraine are common findings in glaucoma patients who were suspected to have systemic vascular dysregulation as a contributing factor in glaucoma [6,7,8]. However, few studies have evaluated the relationship between systemic vascular dysregulation and the ECM properties of the ONH in glaucoma.

The renin–angiotensin–aldosterone system (RAAS) is vital for maintaining arterial BP, retaining extracellular fluid volume and systemic vasoconstriction [9]. Low arterial BP leads to increased production of renin, which hydrolyzes the liver protein angiotensinogen to angiotensin I and II (AngII) [10]. Recent reports have demonstrated that increases in AngII levels, particularly through the AngII type 1 receptor (AT-1R), induce tissue fibrosis in the heart, kidneys, liver, and vessels [11,12,13,14,15]. AngII receptors are expressed in the retina and sclera, and increased expression of AngII activates retinal cells and scleral fibroblasts [16,17]. One study showed that selectively inhibiting AT-1R with losartan decreases activation of the scleral fibroblasts after ocular hypertension and protects the RGCs [18]. There have been recent attempts to modulate the tissue properties of the sclera in experimental glaucoma to protect RGCs. Increasing scleral cross-linking by applying glyceraldehyde leads to increased scleral stiffness and more death of RGCs in an experimental mouse model of glaucoma [19,20,21]. Therefore, the role of the sclera in the pathogenesis of glaucoma is emerging, and there have been attempts to identify the relationship between the tissue properties of the sclera and the loss of RGCs. In addition, modulating the sclera to protect the RGCs is part of another ongoing investigation.

We hypothesized that the RAAS plays a role in glaucoma pathogenesis by influencing the ECM in the ONH region. In particular, glaucoma patients with systemic vascular dysregulation and low or unstable BP may have an activated RAAS [22]. If the activated RAAS induces the activation of scleral fibroblasts and contributes to changes in the ECM, we could propose that systemic vascular dysregulation accompanied by changes in the sclera contributes to the pathogenesis of glaucoma. We previously confirmed a systemic hypotensive rat model characterized to have low and unstable BP using systemic hypotensive medication [23]. Using this systemic hypotensive glaucoma model, we aimed to determine the changes in the scleral ECM and their effect on RGCs under low or unstable systemic BP. First, the changes in the level of AngII receptors and ECM components in the sclera and their effect on the biomechanical properties of the sclera after inducing systemic hypotension were evaluated. Second, the effect of losartan on scleral fibroblasts and its relationship with the AngII receptor after inducing systemic hypotension were investigated. Finally, the effects of losartan on the death of RGCs after modulating the sclera by administering losartan in the sub-Tenon’s tissue were investigated.

## 2. Results

### 2.1. Confirmation of the Systemic Hypotensive Model and Involvement of the RAAS in the Sclera

**Figure 1:**
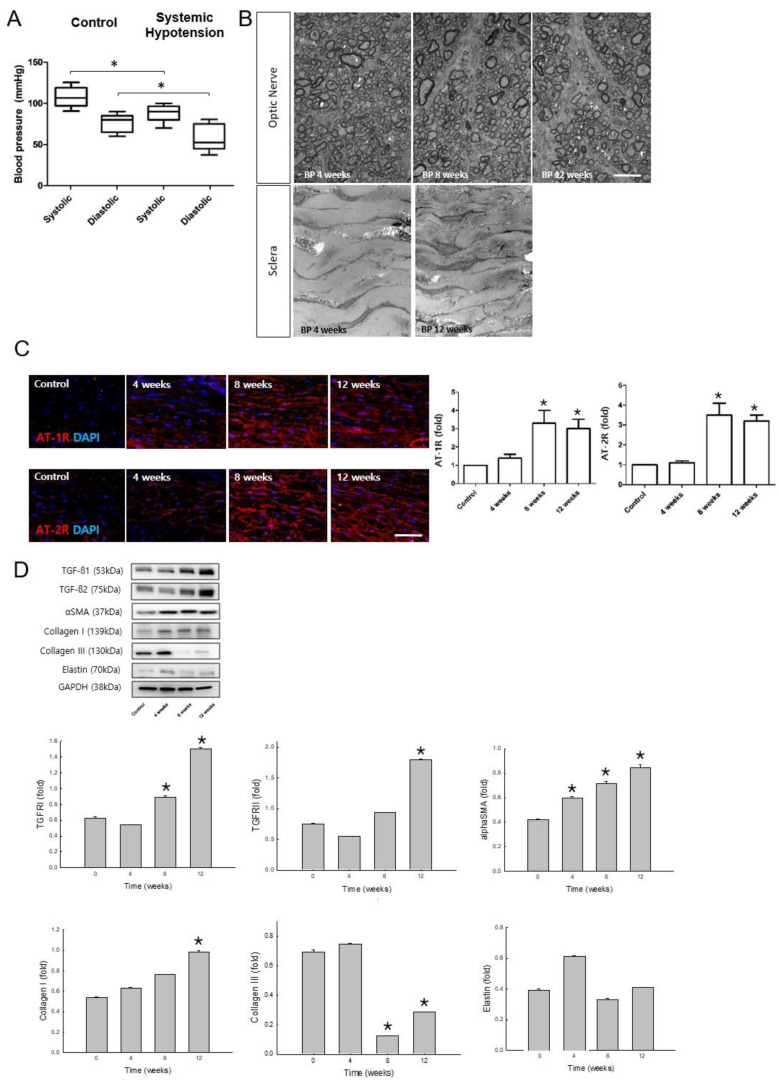
(**A**) Systolic and diastolic blood pressure (BP) of control and systemic hypotensive rats. Rats administered diuretics exhibited lower systemic BP than controls. For BP measurements, n = 6 for control and n = 6 for systemic hypotension; total n = 12. The bar represents mean ± SD. There were statistically significant differences between systolic and diastolic BP of control and systemic hypotension groups, all *p* < 0.001 (**B**) Transmission electron microscopic evaluation of the optic nerve and the sclera of systemic hypotensive rats, n = 3 for each time point; Scale bar = 10 nm. BP decreased in the hypotensive rats throughout the day. (**C**) Immunohistochemical staining of angiotensin II (AngII) receptors. AngII receptor type I (AT-1R; top panel) and type II (AT-2R; lower panel). Significant increases in the expression levels of AT-1R and AT-2R were found in the scleral tissue. For immunohistochemical staining, n = 6 for control and n = 6 for systemic hypotension at each time period; total n = 24; Scale bar = 10 μm. The bar represents mean ± SD. Student’s t-test was used for the statistical evaluation. * *p* < 0.05 compared to the control. (**D**) Western blotting of proteins associated with scleral fibrosis (transforming growth factor-beta [TGF-β] type I and II, alpha-smooth muscle actin [α-SMA], collagen type I and III, and elastin). Protein expression of TGF-β1, TGF-β2, αSMA, and collagen type I increased significantly and that of collagen type III decreased significantly in the sclera after systemic hypotension. For Western blotting analysis, n = 6 for control and n = 6 for systemic hypotension at each time period; total n = 48. The bar represents mean ± SD. Student’s *t*-test was used for the statistical evaluation. * *p* < 0.05 compared to the control.

Animals administered hydrochlorothiazide (HCTZ) exhibited lower and fluctuating systolic and diastolic BPs than the controls at 12 weeks. Tail-cuff-measured systolic and diastolic BPs of the systemic hypotensive rats were 97.3 ± 15.9 and 54.9 ± 20.6 mmHg, respectively. The systolic and diastolic BPs of the control group were 105.5 ± 10.8 and 89.4 ± 9.7 mmHg, respectively (Figure 1A, systolic and diastolic BP between control and systemic hypotension groups, all *p* < 0.001). The SD of the BP measurements was greater in the systemic hypotensive rats than in the control rats, indicating fluctuations in BP (*p* < 0.001).

Changes in the optic nerve and the sclera of the systemic hypotensive rats were investigated using TEM. The optic nerve showed a loss of RGC axons and accumulation of the ECM, indicating progressive axonal damage at 4, 8, and 12 weeks after systemic hypotension (Figure 1B, upper panel). Scleral fibroblasts revealed hypertrophic changes with increased electrodense deposits within and around the fibroblasts, indicating activation of the scleral fibroblasts and accumulation of ECM in the scleral tissues of the systemic hypotensive rats (Figure 1B, lower panel).

**Figure 2:**
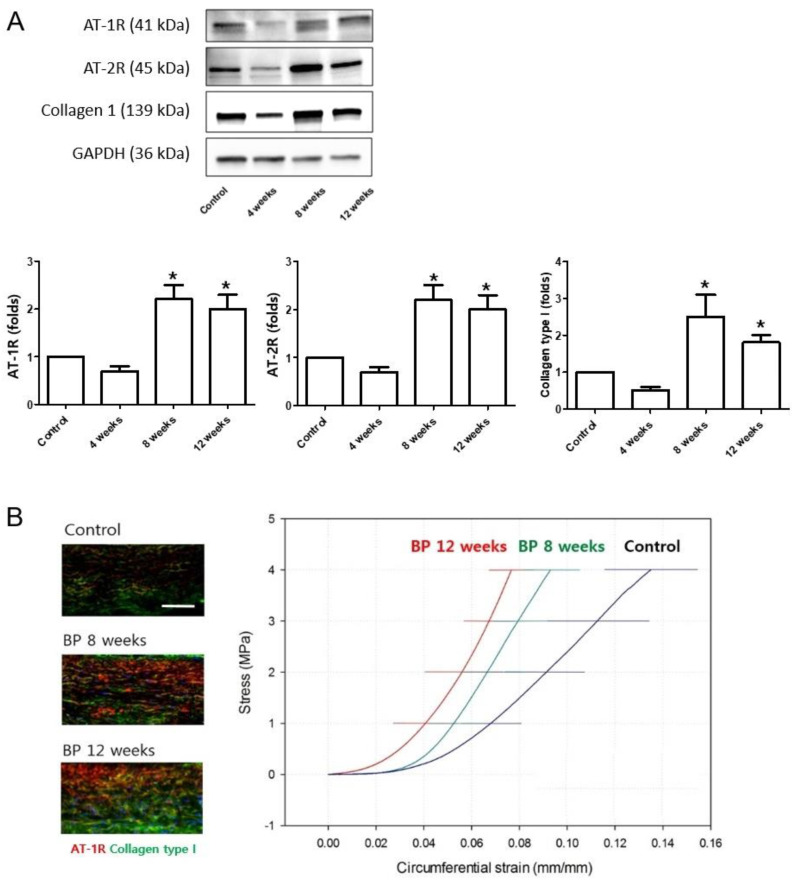
(**A**) Western blotting analysis of the Ang II receptors and collagen type I and the relationship between collagen type I expression and the biomechanical properties of the sclera using stress-strain analysis. Protein expression of the AngII receptors in the sclera; AT-1R and AT-2R increased significantly at 8 and 12 weeks and collagen type I increased at 8 weeks after systemic hypotension. * *p* < 0.05 compared to the control. (**B**) The stress–strain curves in the clinical range of IOP were obtained using scleral strip specimens. As the duration of systemic hypotension was extended, the sclera experienced less strain, indicating less tissue deformation or stiffened tissue under the same load. The decrease in strain was accompanied by increased expression of AT-1R and collagen type I. For stress–strain analysis, n = 6 for control and n = 6 for systemic hypotension at each time period; total n = 24; Scale bar = 10 μm. The bar represents mean ± SD.

To examine the involvement of the RAAS after the reduction and fluctuations in systemic BP, AngII receptor expression was examined in the sclera by immunohistochemical staining. AT-1R and AT-2R expression increased at 8 and 12 weeks compared to the baseline (Figure 1C; all *p* < 0.001). Factors that activate fibroblasts (TGF-β1 and TGF-β2) and indicate transformation to myofibroblasts (α smooth muscle actin [SMA]), as well as the major ECM proteins in the sclera (collagen type I and III, and elastin) were observed in the sclera of systemic hypotensive rats by Western blotting analysis (Figure 1D). The protein expression levels of TGF-β1, TGF-β2, αSMA, and collagen type I increased significantly in the sclera after systemic hypotension. Expression of collagen type III decreased significantly in the sclera 8 and 12 weeks after systemic hypotension. In addition, protein expression of the AngII receptors AT-1R and AT-2R increased significantly in the sclera 8 and 12 weeks after systemic hypotension (Figure 2A; all *p* < 0.001). Therefore, we confirmed that the reduced and unstable systemic BP levels induced increased expression of the AngII receptors in the sclera, and this could be related to the activation of scleral fibroblasts and the accumulation of the ECM in the sclera after systemic hypotension.

### 2.2. Changes in the Biomechanical Properties of the Sclera after Systemic Hypotension

To investigate the mechanical behavior of the sclera after systemic hypotension, stress–strain curves of the scleral strip specimens were obtained. The specimens exhibited nonlinear load elongation and stress–strain behavior, with an initial low tangent stiffness that increased gradually under higher stress. The stress–strain curve was measured between the clinical range of the IOP in the circumferential (parallel with the fiber direction around the ONH) and meridional directions (perpendicular with the fiber direction around the ONH; Figure 2B). As the duration of systemic hypotension increased, the sclera experienced less strain, which means there was less tissue deformation under the same load on the tissue or stiffened tissue. This finding is consistent with the circumferential and meridional directions in all tissues. The decrease in strain was accompanied by increased expression of AT-1R and collagen type I in the same tissue. Taken together, our results demonstrate that the sclera showed a stiffened tissue property or scleral fibrosis that was associated with increased AT-1R expression and ECM components, particularly collagen type I, during systemic hypotension. This was mediated by activation of the scleral fibroblasts, which changed the tissue properties of the sclera.

### 2.3. Involvement of the RAAS and ECM Changes in the Sclera

To confirm that the activated RAAS contributed to the activation of scleral fibroblasts and the accumulation of the ECM in the sclera, we added AngII and losartan, an AngII receptor blocker, to cultured scleral fibroblasts from systemic hypotensive rats at the 4-, 8-, and 12-week time points.

**Figure 3:**
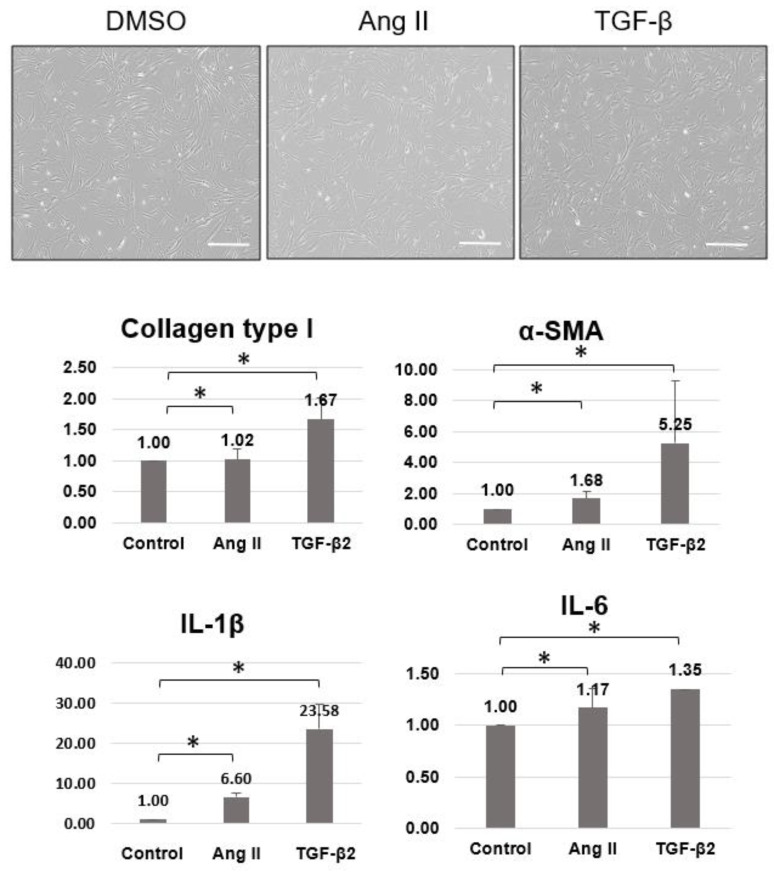
Quantitative PCR of collagen type I, αSMA, and inflammatory cytokines (IL-1β and IL-6) after AngII/TGF-β application to scleral fibroblasts. Compared to controls, collagen type I, αSMA, IL-1β, and IL-6 were significantly increased after AngII and TGF-β application to scleral fibroblasts. For quantitative PCR analysis, n = 3 for each group; total n = 9. Scale bar = 500 μm. The bar represents mean ± SD. * *p* < 0.05 compared to the control.

To compare the role of AngII in the activation of scleral fibroblasts, TGF-β was used as a positive control. The mRNA expression of collagen type I, αSMA, and inflammatory cytokines such as interleukin (IL)-1β and IL-6 were significantly increased with AngII and TGF-β compared to controls (Figure 3).

**Figure 4:**
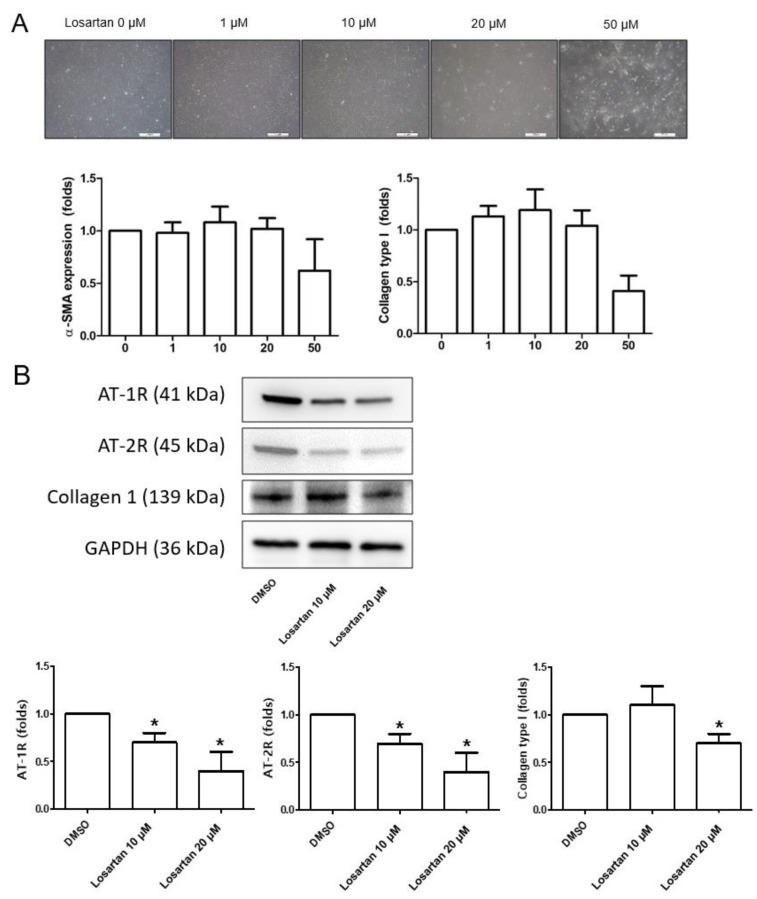
(**A**) Losartan 0~50 μM was applied in a dose-dependent manner to scleral fibroblasts and toxicity started to occur at a concentration of 50 μM in vitro. (**B**) Western blotting of Ang II receptors and collagen type I in the cultured scleral fibroblasts from systemic hypotensive rats. Losartan (10 or 20 μM) was added to cultured scleral fibroblasts. The expression levels of AT-1R, AT-2R, and collagen type I were downregulated in the scleral fibroblasts after applying losartan. We selected Losartan 20 μM from these data for further experiments. Scale bar = 500 μm. The bar represents mean ± SD. Student’s *t*-test was used for the statistical evaluation. * *p* < 0.05 compared to the control.

To determine the concentration of Losartan for further experiments, dose-dependent application of losartan 0~50 μM was performed on the scleral fibroblasts. Toxicity started to occur at a concentration of 50 μM in vitro (Figure 4A). The expression of AT-1R and AT-2R in the sclera was downregulated after adding losartan 10 or 20 μM to the culture media of the scleral fibroblasts from systemic hypotensive rats at 12 weeks (Figure 4B, all *p* < 0.001). In addition, collagen type I expression decreased significantly after the losartan 20 μM treatment (*p* < 0.001). From these data, we choose 20 μM of losartan for further in vitro studies.

**Figure 5:**
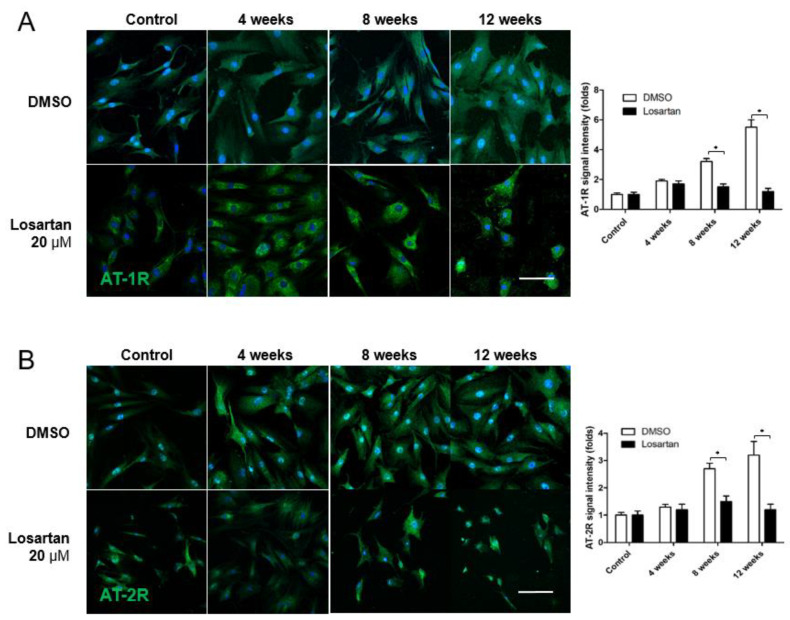
(**A**,**B**) Immunocytochemical staining of AT-1R and AT-2R. AT-1R and AT-2R expression showed gradual increase in the cultured primary scleral fibroblasts on immunocytochemistry 4, 8, and 12 weeks after systemic hypotension. Applying losartan 20 μM significantly decreased the expression of the AT-1R and AT-2R 4, 8, and 12 weeks after systemic hypotension. Scale bar = 200 μm. * *p* < 0.05 compared to the control.

**Figure 6:**
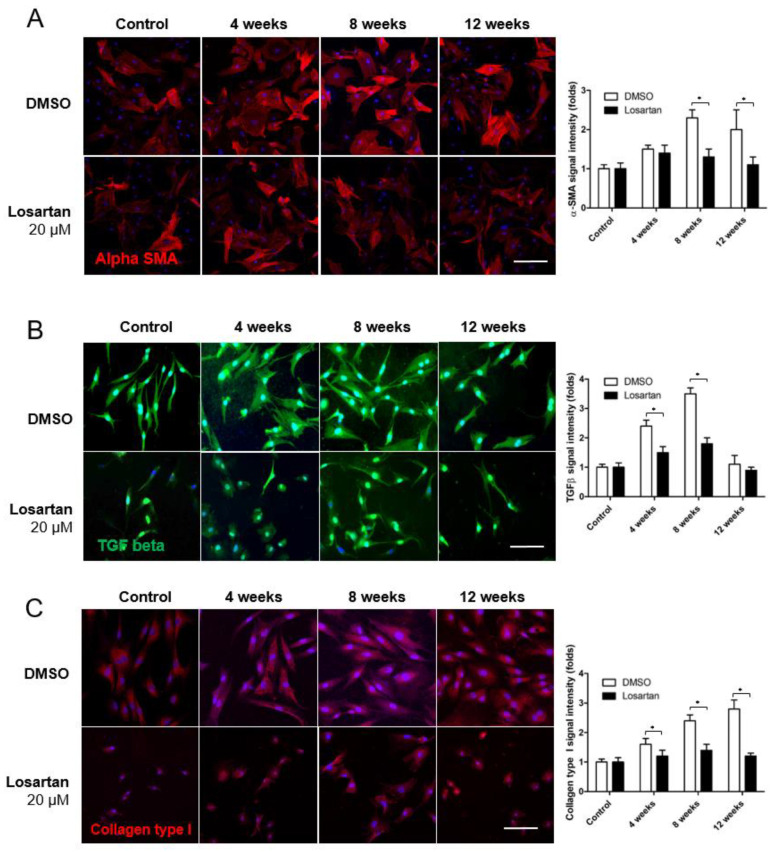
(**A**–**C**) Immunocytochemical staining of α-SMA, TGF-β, and collagen type I. The expression of α-SMA and TGF-β, which shows the activation of scleral fibroblasts, increased in response to systemic hypotension and was downregulated by the losartan 20 μM treatment. The expression level of collagen type I also increased in response to systemic hypotension but decreased in response to the losartan treatment. Scale bar = 200 μm. * *p* < 0.05 compared to the control.

The expression of AT-1R and AT-2R increased gradually in the primary scleral fibroblast culture on immunocytochemistry 4, 8, and 12 weeks after systemic hypotension (Figure 5A,B). Applying 20 μM losartan significantly decreased the expression of AT-1R and AT-2R at each time point after systemic hypotension. The expression of αSMA and TGF-β, which activate scleral fibroblasts, increased in response to systemic hypotension (Figure 6A,B) and was downregulated by the losartan treatment. The expression level of collagen type I also increased in response to systemic hypotension, which was reduced by the losartan treatment (Figure 6C). AngII receptor expression was downregulated by treating the scleral fibroblasts with losartan, which decreased their activation and production of ECM.

### 2.4. Effect of Inhibiting Angiotensin on RGCs

**Figure 7:**
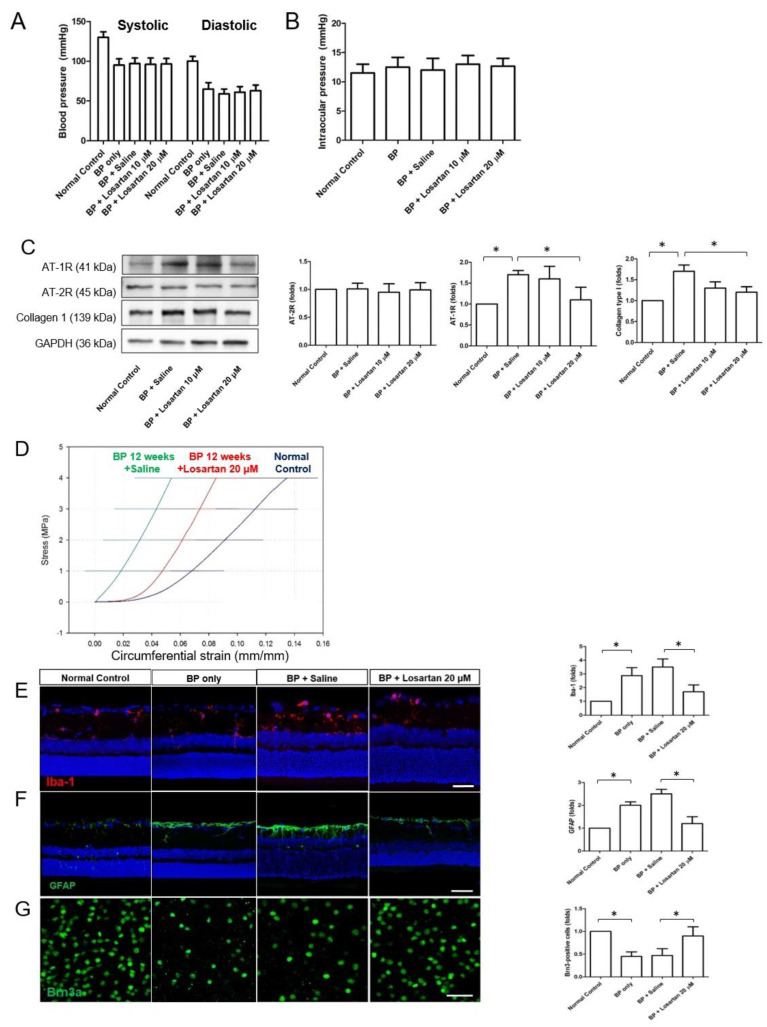
(**A**,**B**) Blood pressure and intraocular pressure of normal control, hypotension model (BP only), after sub-Tenon injection of saline, losartan 10 μM, and 20 μM. (**C**) Western blotting of Ang II receptors and collagen type I in normal control and after sub-Tenon injection of saline and losartan in the systemic hypotensive rats. The expression levels of AT-1R and collagen type I, but not AT-2R, were downregulated in the sclera after sub-Tenon injection of losartan in the systemic hypotensive rats. For Western blotting analysis, n = 6 for control and n = 6 for saline and each concentration of losartan; total n = 24. The bar represents mean ± SD. Student’s t-test was used for the statistical evaluation. * *p* < 0.05 compared to the control. (**D**) The stress–strain curve for the biomechanical analysis. The lower strain in the sclera observed after 12 weeks of systemic hypotension increased after the sub-Tenon injection of losartan treatment. For stress–strain analysis, n = 3 for control and n = 6 for systemic hypotension with and without losartan treatment; total n = 9. The bar represents mean ± SD. (**E**–**G**) Iba-1 and GFAP immunohistochemical staining of glial cells and Brn3a for RGCs. A significant decrease in microglial and macroglial activation was noted in the retina of systemic hypotensive rats after treating the sclera with losartan. A significant increase in the number of RGCs was noted after treating the sclera with losartan. For immunohistochemical staining, the number of rats were: n = 6 for control and, n = 6 for systemic hypotension only without treatment, n = 6 for sub-Tenon injection of saline and lorsartan, total n = 24; (**E**,**F**) Scale bar = 100 μm; (**G**) Scale bar = 50 μm. The bar represents mean ± SD. Student’s *t*-test was used for the statistical evaluation. * *p* < 0.05 compared to the control.

We administered losartan to systemic hypotensive rats at the 12-week time point. Both 10 and 20 μM doses of losartan that were not toxic and had an effect on scleral fibroblasts were used in animal studies. Administering losartan as sub-Tenon injection did not affect the systemic BP and IOP (Figure 7A,B). The expression levels of AT-1R and collagen type I in the scleral tissue were upregulated after systemic hypotension compared to those of normal rats (Figure 7C). The expression levels of AT-1R, but not AT-2R, were downregulated in the sclera of the systemic hypotensive rats after the sub-Tenon injection of 20 μM losartan (Figure 7C, all *p* < 0.001). In addition, collagen type I expression decreased significantly after 20 μM losartan treatment (*p* < 0.001). Therefore, we used 20 μM losartan for further animal studies and confirmed that administering losartan downregulated AT-1R, and the ECM component collagen type I decreased in the sclera. The stress–strain curve was measured after treatment (Figure 7D). Lower strain was detected in the sclera after 12 weeks of systemic hypotension after the losartan treatment, indicating less stiffening of the sclera, which correlated with the ECM changes observed during Western blotting analysis.

To determine the effect of inhibiting angiotensin and changes in the ECM on the RGCs, Iba-1 and GFAP immunohistochemical staining was performed, and it revealed activation of the glial cells following systemic hypotension in the retina compared to normal rats (Figure 7E,F). However, significant decreases in microglial and macroglial activation were noted in the retina of the systemic hypotensive rats after the losartan treatment. To examine the changes after treatment, we performed Brn3a immunohistochemical staining in flat-mounted retinas. A decrease in the number of RGCs was observed following systemic hypotension compared to normal retina (Figure 7G). However, a significant increase in the number of RGCs was noted after losartan treatment. These findings suggest that inhibition of the expression of angiotensin receptors in the sclera by losartan reduced the activation of glial cells and protected against the loss of RGCs caused by systemic hypotension.

## 3. Discussion

This study explored the effects of RAAS on tissue properties of the sclera and its role in the death of RGCs in glaucoma. We previously confirmed that low and unstable BP upregulates the AngII level in the serum and retinal tissues of this systemic hypotensive animal model and that this was related to the death of RGCs by necroptosis [23]. As AngII has a role in tissue fibrosis of various organs, AngII could play a role in scleral fibrosis. Increased AT-1R and AT-2R levels were detected in the sclera after inducing systemic hypotension in the animal model. The increase in the AngII receptors triggered changes in the ECM of the sclera, resulting in upregulation of collagen type I and downregulation of collagen type III/elastin, which increased the tensile strength of the sclera. These findings were confirmed in an in vitro model of systemic hypotension in which scleral fibroblasts from systemic hypotensive rats were cultured. Inhibiting AngII with losartan downregulated the AngII receptors and collagen type I in the sclera and this protected against loss of RGCs after systemic hypotension. These findings suggest that AngII has a role in scleral fibrosis after systemic hypotension and that inhibiting AngII may modulate the tissue properties of the sclera to protect the RGCs. Decreasing scleral stiffening by modulating the ECM components and inhibiting AngII protected against glaucomatous damage caused by systemic hypotension.

The sclera, which is a continuum of the cornea and the lamina cribrosa, constitutes the outer coating of the eye and plays a role in supporting the neurons in the retina and the optic nerve. Weakness in the outer coat tissues of the eye contributes to the loss of RGCs under normal range IOPs in this situation [24,25,26]. These findings have generated increased interest in the biomechanical properties of the ocular coat, including the sclera and its role in the pathophysiology of glaucoma. The sclera changes substantially in experimental models of glaucoma, and these changes are reported to affect the susceptibility to glaucomatous damage or associated loss of RGCs [27,28]. Proteomic analysis of the sclera indicates activation of TGF and activators of scleral fibroblast during the glaucomatous process [29,30]. We previously reported an animal model with characteristics of glaucoma and an unstable BP, but with a normal range IOP [23]. These systemic hypotensive rats were designed to induce an imbalance in the RAAS and destabilize hemodynamics. Since AngII receptors have been identified in the sclera, we investigated the role of AngII in scleral fibrosis and the contribution to the loss of RGCs after systemic hypotension. As expected, scleral fibrosis occurred after upregulation of AngII receptors in the sclera and induced ECM changes in the sclera in our experiment. Modulating this process had a protective effect on the survival of RGCs after systemic hypotension.

Tissue fibrosis caused by AngII acts mainly through AT-1R. AT-1R is expressed in the sclera, corneal myofibroblasts, and anterior ocular fibroblasts and responds to AngII, resulting in fibrogenesis [18,31]. The RAAS activates TGF-β, and local RASs have been identified in several tissues, including the eye [32,33,34]. AT-1R signaling upregulates thrombospondin, a potent activator of TGF-β, which decreases after inhibiting the AT-1R with losartan [18]. In the present study, systemic hypotension induced scleral stiffening mediated by the AngII receptor. Inhibiting the AT-1R with losartan downregulated TGF-β in scleral tissues and scleral fibroblasts from systemic hypotensive rats. This affected the composition of the ECM and the tissue properties of the sclera, resulting in less scleral stiffening and protection against the loss of RGCs caused by systemic hypotension. Therefore, we confirmed that AngII mediates scleral fibrosis, and it contributed to the loss of RGCs in glaucoma after systemic hypotension. Modulating AngII with losartan has the potential to protect against the loss of RGCs caused by systemic hypotension. Previous studies have shown that inhibiting AngII has a neuroprotective effect in various glaucoma models, such as ocular hypertension, ischemia–reperfusion, and axotomized retinal explants [35,36,37]. Inhibiting AngII had a neuroprotective effect in the systemic hypotensive model and its effect occurred through scleral tissue remodeling. The role of scleral tissue properties and biomechanics in glaucoma pathogenesis is complex, and more investigations are needed. The present data support the importance of the scleral properties in damage to RGCs after hemodynamic instability, and scleral remodeling seems to be important in preventing the loss of RGCs in glaucoma.

## 4. Materials and Methods

### 4.1. Animals

All experiments were performed following the Association for Research in Vision and Ophthalmology statement for the Use of Animals in Ophthalmic and Vision Research. We also followed the National Institutes of Health Guide for the Care and Use of Laboratory Animals (NIH Publications, no. 80–23, revised 1996). All animals were cared for according to the regulations of the Ethics Committee of the Catholic University and the Institutional Animal Care and Use Committee of the Catholic University of Korea. A total of 104 animals were used. Careful management of the animals and procedures was ensured to minimize the number of animals used.

A sub-Tenon injection (29-gauge needle) of 0.10 mL losartan (20 μM; Cozaar, Merck, Whitehouse Station, NJ, USA) in saline was administered 3.0 mm behind the limbus in the inferonasal and superotemporal quadrants of the eye. A small-incision hook was used to spread the solution to the back of the eyeball. All injections were administered under inhalation anesthesia. A sub-Tenon injection of 0.10 mL saline was administered to the control group using the same strategy. Daily antibiotic eye drops were applied 3 days after the procedure.

### 4.2. Systemic Hypotensive Rats

Adult male Sprague–Dawley rats (weight, 250–300 g; age, 7–8 weeks) were used in this study. The systemic hypotensive rat model was designed using HCTZ (Dichlozid; Yuhan, Seoul, Korea; 100 mg/kg dissolved in drinking water) to lower systemic BP without interfering with the Ang pathways [23,38]. The animals were randomly assigned to either the experimental or control groups. Systolic and diastolic BP were measured using a tail cuff sphygmomanometer (Visitech BP2000, Visitech Systems, Apex, NC, USA) twice daily at the same time [39,40]. Measurements were obtained every week throughout the 12-week experimental period, and the mean value of the two measurements was calculated. The time and frequency of water drinking by individual rats were irregular, which could induce BP fluctuations. Rats without a significant reduction in systolic BP < 120 mmHg or diastolic BP < 80 mmHg were excluded (7 of 104 animals; 6.7%). The standard deviations (SDs) of all measurements were calculated and the mean SD of each group was considered the BP fluctuation.

### 4.3. Immunohistochemistry of the Sclera and Retina

After the rats were sacrificed, both eyes were enucleated and fixed in 4% paraformaldehyde at 4 °C for 10 min. The anterior segment of the eye was removed, and the posterior segment was fixed in 4% paraformaldehyde for 60 min. The scleral and retinal tissues were embedded in OCT compound and sectioned to a thickness of 6 μm by cryosectioning. After washing several times with phosphate-buffered saline (PBS), non-specific binding was blocked with 10% normal donkey serum in PBS for 2 h at room temperature. The slides were incubated overnight at 4°C with the following primary antibodies. Mouse anti-brain-specific homeobox/POU domain protein 3a (anti-Brn3a; Millipore, Billerica, MA, USA, 1:200), glial fibrillary acidic protein (GFAP; Millipore, 1:200), ionized calcium-binding adapter (Iba-1; 1:200), AngII receptor type 1 (AT-1R) (1:100, Abcam, Cambridge, MA, USA), AngII receptor type 2 (AT-2R) (1:100, Abcam), and collagen type I (1:100, Abcam) were used to observe the AngII receptors in the sclera and glial activation/RGC death in the retina. Primary antibody binding was detected by Alexa488- and Alexa546-conjugated secondary antibodies (Molecular Probes, Eugene, OR, USA). The slides were rinsed in 1× PBS and mounted with Fluoroshield mounting media including DAPI (Vector Laboratories, Burlingame, CA, USA). Images of these stained tissues were acquired using confocal laser scanning microscopy (Carl Zeiss, Jena, Germany).

### 4.4. Transmission Electron Microscopy (TEM)

The optic nerve and scleral tissues were fixed by immersion in Kamovsky’s solution for 24 h at 4 °C and then embedded in acrylic resin. Ultrathin sections (0.1 μm) were prepared, mounted on Formvar-coated slot grids, and stained with 3% lead citrate. The TEM images were acquired using a Zeiss transmission electron microscope (Zeiss Inc., Thornwood, NY, USA).

### 4.5. Biomechanical Analysis

Rectangular scleral tissue strips (4.0 mm width and 6.0 mm length) were extracted in the circumferential and meridional directions. The thickness and width of each strip were measured using high-frequency B-mode ultrasound (Vevo660, VisualSonics, Toronto, ON, Canada). The strips were stored in PBS solution at 4 °C before testing. All mechanical testing was performed within 4 h of excising the strips. The stress–stain relationship was measured using the Biomaterial Universal testing machine (Instron Model 5966, Illinois Tool Works, Grove City, PA, USA) at room temperature with a 50 N load. The 6.0 mm length strips were clamped at 2.0 mm on each side. The distance between the clamps was 2 mm. Force and length data were collected automatically by a computer. This test produces axial load and elongation results. Stress was calculated by dividing the applied load by the mean cross-sectional area (thickness × width) of the specimen. Strain was calculated by dividing the specimen length by the initial measured length. The measured scleral tissue underwent immunohistochemical staining against AT-1R and collagen type I.

### 4.6. Fibroblast Culture

Rat eyes were enucleated and scleral tissues were obtained from the control, and 4, 8, and 12 weeks after systemic hypotension. A 2 mm wide scleral band surrounding the ONH was isolated and cut into 1 × 1 mm sections. The sclera pieces were placed on 35 mm collagen-coated, tissue culture dishes containing RPMI-1640 medium, 20% fetal bovine serum (FBS), nonessential amino acids, 1% penicillin/streptomycin, and sodium pyruvate. After 14 days, the cells were passaged in Dulbecco’s modified Eagle medium with 10% FBS, 1% penicillin/streptomycin, and sodium pyruvate. All experiments were conducted on cells between passages 3 and 5. After performing a dose-dependent response using AngII and TGF-β, 1 μM of AngII and 5 ng/mL of TGF-β were applied to the culture media. Losartan was applied to cultured cells at a concentration of 10 or 20 μM.

### 4.7. Quantitative Real-Time Polymerase Chain Reaction (RT-PCR)

Total RNA was extracted from scleral fibroblast using the RNeasy kit (Qiagen, Hilden, Germany), including an on-column DNase I digestion step. First-strand cDNA synthesis from 0.1 μg of total RNA and quantitative RT-PCR were performed using the MyIQ thermal cycler and software (Bio-Rad Laboratories, Munich, Germany). PCRs (25 μL), run in duplicate, contained 2 μL of first-strand cDNA, 0.4 μM each of upstream and downstream primers (Macrogen, Inc., Seoul, Korea), and IQ SYBR Green Supermix (Bio-Rad Laboratories). The primers and PCR conditions are as follows; Collagen type I (Forward, GGCTACTTCTCGCTCTGCTTCATC; Reverse, TGGGCAAACTGCACAACATTCTCC), αSMA (Forward, CTATGCCTCTGGACGCACAACT; Reverse, CAGATCCAGACGCATGATGGCA), IL-1β (Forward, CCACAGACCTTCCAGGAGAATG; Reverse, GTGCAGTTCAGTGATCGTACAGG), IL-6 (Forward, AGACAGCCACTCACCTCTTCAG; Revese, TTCTGCCAGTGCCTCTTTGCTG), and GAPDH (Forward, AGCTCACTGGCATGGCCTTC; Reverse, ACGCCTGCTTCACCACCTTC) summarized in Table. For quantification, serially diluted standard curves of plasmid-cloned cDNA were run in parallel, and amplification specificity was checked using melt curves and sequence analyses on the CPX96 Real Time PCR Machine (Bio-Rad Laboratories). mRNA ratios relative to the housekeeping gene GAPDH were calculated to normalize gene expression levels. For each analyzed tissue, three samples were pooled in each group.

### 4.8. Immunocytochemistry

Scleral fibroblasts were seeded in 24-well plates containing coverslips coated with 0.1% gelatin. The cells were fixed at room temperature for 15 min in 4% formaldehyde/PBS and washed twice with PBS, followed by permeabilization with 0.1% Triton X-100 in PBS for 10 min and blocking with 5% bovine serum albumin (BSA; Sigma-Aldrich Corp., St. Louis, MO, USA) in PBS for 1 h. Diluted primary antibodies (AT-1R, AT-2R, αSMA, collagen type I, and TGF-β1) in 1% BSA were incubated overnight at 48°C. The secondary antibodies (Alexa488 and Cy3; Thermo Fisher Scientific, Waltham, MA, USA) were incubated for 1 h at room temperature. All images were captured by confocal fluorescence microscopy (Leica SP8; Leica, Berlin, Germany).

### 4.9. Western Blotting Analysis

Scleral tissues and cells were lysed in radioimmunoprecipitation assay (RIPA) buffer [50 mM Tris-HCl pH 7.5, 150 mM NaCl, 1 mM EDTA, 0.1% SDS, 1% IGEPAL and 0.5% sodium deoxycholate] containing protease and phosphatase inhibitor cocktails. The lysates were clarified by centrifugation at 10,000× *g* for 25 min at 4 °C. The supernatants were assayed to measure protein content using a standard bicinchoninic acid assay (Pierce, Rockford, IL, USA). Retinal extracts were separated by SDS-polyacrylamide gel electrophoresis and transferred onto a nitrocellulose membrane (Hybond-C, Amersham Pharmacia Biotech, Mannheim, Germany). The blots were stained with Ponceau S (Sigma) to visualize the protein bands and to ensure equal protein loading and uniform transfer. The membranes were blocked for 45 min with 5% non-dried skim milk in Tris-buffered saline with Tween buffer (20 mM Tris-HCl pH 7.6, 137 mM NaCl, and 0.1% Tween20). Then, the blots were probed for 24 h with antibodies against AT-1R (Abcam), AT-2R (Abcam), TGF-β1 (Abcam), TGF-β2 (Abcam), αSMA (Sigma), collagen type I (Abcam) and type III (Abcam), elastin (Elastic Products Co, MO, USA), and GAPDH (Sigma). The blots were sequentially probed with horseradish–peroxidase-conjugated goat secondary antibody for 1 h at room temperature. The protein bands were detected using an enhanced chemiluminescence system (Amersham, Woburn, MA, USA) and X-ray film. The relative intensity of the blots was measured using ImageMaster VDS (Pharmacia Biotech, City of Industry, CA, USA), and the fold-changes in protein levels were calculated relative to GAPDH.

### 4.10. Immunohistochemistry of Flat-Mounted Retinas

Intact retinas were extracted and washed in PBS after fixation in 4% paraformaldehyde. Anti-Brn3a was used as the primary antibody to label the RGCs on the retinal flat mounts. The immunostained retinas were carefully flattened and mounted on microscopic slides with Fluoromount (Southern Biotech, Birmingham, AL, USA) and imaged using confocal laser scanning microscopy (Carl Zeiss).

Each flat-mounted retina was divided into four equal quadrants. Three fields measuring 200 × 250 μm^2^ were randomly sampled from the middle regions of each retinal quadrant. The distance to the optic nerve from the corresponding field of each quadrant was equal. Labeled ganglion cells were counted at 200× magnification in 12 regions of each retina.

### 4.11. Statistical Analysis

All data are presented as means ± SDs. The two-sided Student’s *t*-test was used to compare the controls and experimental animals at each time point. A *p*-value < 0.05 was considered significant.

## 5. Conclusions

In conclusion, this study demonstrated that systemic hypotension could trigger AngII in the sclera, resulting in scleral fibrosis. This may lead to a loss of RGCs after systemic hypotension by changing the composition of the ECM and the tensile strength of the sclera. Therefore, hemodynamic instability could contribute to progression in glaucoma patients by inducing changes to the sclera. Modulating AngII, particularly the AT-1R, was associated with changes in ECM expression and the tissue properties of the sclera following systemic hypotension, and this protected against loss of RGCs. These results highlight the need to identify the causes of RGC loss related to the sclera and the hemodynamic features of glaucoma to optimize the management of glaucoma and personalize treatment strategies to prevent glaucoma-associated blindness.

## Data Availability

Data may be provided upon reasonable request.

## Abbreviations

AngII	Angiotensin II
AT-1R	Angiotensin II receptor type I
AT-2R	Angiotensin II receptor type II
ECM	Extracellular matrix
HCTZ	Hydrochlorothiazide
IL	Interleukin
ONH	Optic nerve head
RAAS	Renin–angiotensin–aldosterone system
RGC	Retinal ganglion cell
SMA	Smooth muscle actin
TGF	Transforming growth factor
